# Receiving Return-To-Work Coordination at Swedish Primary Healthcare Clinics: A Register-Based Study Among People on Sickness Absence

**DOI:** 10.1007/s10926-025-10330-2

**Published:** 2025-09-03

**Authors:** Erik Berglund, Emilie Friberg, Veronica Svärd

**Affiliations:** 1 Division of Insurance Medicine, Department of Clinical Neuroscience, Karolinska Institutet, 171 77 Stockholm, Sweden; 2 Department of Public Health and Caring Sciences, Uppsala University, 751 22 Uppsala, Sweden; 3 Department of Social Work, School of Social Sciences, Södertörn University, 141 89 Huddinge, Sweden

**Keywords:** Sick leave, Vocational rehabilitation, Return-to-work (RTW), RTW coordinator

## Abstract

**Purpose:**

This study investigates factors associated with receiving return-to-work (RTW) coordination within primary healthcare and the association between received RTW coordination and the amount of sickness absence (SA) days.

**Methods:**

This register study included 13019 people who had SA at some time between March 2019 and February 2020 and visited a primary healthcare clinic that employed a RTW coordinator. Logistic regressions were used to investigate received RTW coordination. Negative binomial regressions were used to analyse the amount of SA days in the ten-month follow-up period.

**Results:**

Associations were found between receiving RTW coordination and being a woman, being 30–49 years old, and having higher educational attainment, more healthcare contacts, more SA days, and a musculoskeletal or mental diagnosis. In the following ten-month period, mean SA were 71.3 days among people receiving RTW coordination and 76.1 days for those who had not. However, the adjusted analyses did not show association between RTW coordination in primary healthcare and the amount of SA days in the following ten-month period.

**Conclusion:**

RTW coordination seems to be directed towards some groups of people with a higher risk of long-term SA, but not all, which raises the question of whether or not more people on SA would have benefited from RTW coordination as well. This would likely mean that more finely calibrated ways of identifying people at risk of long-term SA are needed. The reduction in SA days for those who had received RTW coordination needs to be confirmed in further studies.

## Introduction

Sickness absence (SA) involves considerable social and economic costs, both for the individual and society, especially in the case of long-term SA [1]. The return-to-work (RTW) process often comprises numerous phases, environments, systems, interventions, and interactions that are spread out across multiple stakeholders [2]. For a successful RTW process, RTW coordination is often recommended for improving collaboration and coordination between the individual on SA and different stakeholders, such as healthcare professionals, employers, the Social Insurance Agency, and social services [3, 4]. RTW coordination is intended to facilitate these interactions, by coordinating and providing individualised support for people on SA, and may contribute to establishing a plan for the RTW process [5, 6]. Although RTW coordination is believed to both create and maintain a working alliance between stakeholders in the RTW process [4], research findings are not unanimous about its effect on RTW outcomes. Some studies demonstrate that RTW coordination has a positive effect on RTW [7–9], while others have found no or adverse effects [10, 11].

Many countries have implemented return-to-work coordinators (RTWC) to provide qualified RTW coordination. In most countries, the RTWCs are situated in workplaces or insurance companies [12], but in Sweden, the RTWCs (also called rehabilitation coordinators) have been placed in public healthcare settings for roughly a decade. Since 2020, healthcare clinics in Sweden are obliged to offer patients on SA a coordination of their rehabilitation and RTW processes, if needed [13]. RTW coordination may be provided by other healthcare professionals as well, but it is mainly the responsibility of a RTWC, if one is available.

Very little is known about which groups of patients receive RTW coordination in the Swedish primary healthcare setting. RTWCs in primary healthcare stated in one focus group study that nearly all of their patients had common mental disorders [14]. Many of them also said that they worked with patients with multimorbidity and psychosocial difficulties, even though they argued that these cases took up too much of their time and were not really their responsibility. A survey of RTWCs indicates that primary care RTWCs did not prioritise patients who had been on SA for more than 180 days or who had been denied SA [15]. A questionnaire study of 274 patients who had had contact with a RTWC shows that the average patient age was 47 years and that 79.6% were women and 18.6% men [5]. A majority (62.1%) reported two or more diseases, of which the most common were burnout/fatigue symptoms (24.8%), other mental disorders or neuropsychiatric disorders (37.2%) and pain disorders (8.6%), followed by a range of primarily somatic disorders, injuries, disabilities, or other symptoms.

However, self-reported data might not provide a full picture of the spectrum of individuals receiving RTW coordination. To better understand the patient groups receiving RTW coordination from a primary care RTWC, this study uses the register data of people on SA who visited a primary care clinic in Sweden. This study aimed to investigate factors associated with receiving RTW coordination within primary care settings from March 2019 to February 2020, and the association between received RTW coordination and the number of accumulated SA days in the following ten-month period. The factors we investigated for their association with receiving RTW coordination or not, were demographic factors, type of diagnosis, amount of healthcare contacts, and previous SA days.

## Methods

This study was based on register data from March 2019 to December 2020.

### Study Population and Data Sources

This study included people in Region Stockholm who had at least one period of 90 full or part-time SA days and were in contact with a primary healthcare clinic that had an RTW coordinator in place from March 2019 to February 2020 (65 clinics). This time period was chosen for two reasons. Firstly, in March 2019, RTWCs started to more systematically register RTW coordination in patient records. Secondly, in March 2020 and later, as a consequence of the outbreak of the COVID-19 pandemic, healthcare visits decreased and the regulations for sickness certification were temporarily changed.

The study was based on register data obtained from three government authorities. All of the primary healthcare data from the Region Stockholm regional healthcare data warehouse (VAL database) was obtained. VAL contains information about reported intervention codes based on the Swedish Classification of Health Interventions (Sw. *Klassifikation av vårdåtgärder* (KVÅ)). The codes in the Swedish Classification of Health Interventions are based on those in the International Classification of Health Interventions (ICHI). Data were also obtained from the Micro Data for Analysis of the Social Insurance register (MiDAS) kept by the Social Insurance Agency in Sweden. Data was collected about SA, and disability pension (DP) (2019–2020) paid out by that Agency, including information on diagnoses according to the International Classification of Diseases 10 (ICD-10). Demographic information was obtained from the Longitudinal Integration Database for Health Insurance and Labour Market Studies (LISA) (2019–2020), kept by Statistics Sweden [16]. Individual-level data from the registers were linked using the unique personal identity numbers issued to all residents in Sweden.

The inclusion criteria were: People having at least one period of 90 full or part-time SA days during 2019 or 2020, of which at least one of these SA days was within the period from March 2019 to February 2020; having at least one primary healthcare contact in Region Stockholm during the same period; being between 18 and 65 years of age; and, living in Region Stockholm. People with DP from March 2019 to February 2020 were excluded because RTW coordinators are not supposed to work with this group. However, since some of those included might have been granted DP later, DP was included in the follow-up period. Application of these criteria resulted in a study group of 13,019 people.

### Variables Used

#### Sociodemographic Characteristics

Demographic information was obtained from the LISA database. The demographic data used in this study were sex (female and male), age in 2020 (grouped into; up to 29, 30–39, 40–49, 50–59 and 60–65), educational level (categorised as compulsory or secondary school and university), and country of birth (categorised as Sweden, rest of the Nordic countries, rest of Europe, or rest of the world).

#### Diagnoses, RTW Coordination, and Healthcare Contacts

Information about primary diagnosis was based on the initial medical diagnosis (disease, illnesses, or disabilities) registered in the sickness certificates issued between March 2019 and February 2020. Sickness certificates may include several medical diagnoses as the cause for SA, but the register data only contains information on the primary diagnosis. Primary diagnosis was categorised based on the ICD-10 system as either Mental, Behavioural and Neurodevelopmental disorders; Diseases of the Musculoskeletal system and Connective tissue; or Other (which includes all other types of medical diagnoses). In the following, these will be referred to as mental disorders, musculoskeletal disorders, or other.

Using the Swedish Classification of Health Interventions code for RTW coordination—GC001 (Coordination interventions to promote return to or entry into working life)—documented interventions of received RTW coordination from March 2019 to February 2020 were identified and assessed [17]. Because both RTWCs and other healthcare professionals may deliver RTW coordination, and thus use the intervention code GC001, and as the RTWC is not registered as a profession in the administrative healthcare systems, it was not possible to distinguish if all interventions were indeed performed by a RTWC. However, it is unusual that other professions use the code GC001. As a precaution, this study only included clinics with an employed RTWC. A list of clinics with an employed RTWC during autumn 2019 was compiled through the regional network for RTWCs in primary care. This list was also used for conducting a survey study among RTWCs, the results of which have been published previously [15].

The number of healthcare contacts was based on the number of contacts that each individual made from March 2019 to February 2020 that concerned healthcare interventions other than RTW coordination.

#### Sickness Absence and Disability Pension Measures

People’s inability to work can lead to the need for SA, but also to DP if all possibilities for rehabilitation have been considered and the inability to work is predicted to last for several years. In this study, both SA and DP were analysed. All-cause SA was investigated in the first study period, from March 2019 to February 2020, and all-cause SA as well as DP were investigated in the follow-up period, from March 2020 to December 2020. SA can be granted to residents in Sweden aged 16 and older, either in paid work or receiving unemployment benefits, if their work capacity is reduced due to disease or injury [18]. DP can be granted to residents in Sweden aged 19–64 if a disease or injury leads to a long-term or permanent reduction of work capacity. Both SA and DP can be granted for full-time or part-time, and part-time SA and DP can be received simultaneously. Gross days of SA and DP in the register were transformed into net days, with two gross days of 50% were calculated as one net day per individual. The analyses use 90 days as the cut point for long-term SA since the Swedish evaluation of work capacity within the rehabilitation framework starts at day 90.

Based on the registry data, dates obtained for SA and DP were used for estimating both the amount of net SA days for the time period March 2019 to February 2020 and the amount of net SA and SA/DP days in the ten-month follow-up period were calculated per individual. SA and SA/DP days in the ten-month follow-up period were used as outcomes.

### Analyses

The data were analysed using Chi-square tests to compare differences in distributions and ANOVA for differences between mean values, between study participants who had received RTW coordination and those who had not. Binary logistic regression models were used to compute Odds Ratios (OR) and 95% confidence intervals (CI) for associations between the received RTW coordination, sex, age, education level, country of birth, primary diagnosis, number of healthcare contacts, and SA days. Both crude and adjusted logistic regression analyses were carried out. The adjusted models included sex, age, education level, country of birth, primary diagnosis, number of healthcare contacts, and SA days.

ANOVA was used for analysing differences between mean values in amount of SA, DP and SA/DP between study participants who had received RTW coordination and those who had not. The amount of SA and DP were measured in net days, which is a count variable. Negative binomial regression is suitable for count data and outcome variables with high variance [19]. For the negative binomial regression, decimals in SA and DP net days were rounded to the nearest whole number. Both unadjusted and adjusted negative binomial regression with log link were used. Adjusted models included RTW coordination, sex, education level, country of birth, primary diagnosis, number of interventions in the primary healthcare clinic and amount of SA days from March 2019 to February 2020. The negative binomial regressions produce an incident rate ratio (IRR) for a predictor (where IRR > 1 indicates that the variable is associated with more days). All tests were two-sided and a level of *p* < 0.05 was considered statistically significant. The statistical analyses were performed using SPSS statistics (IBM Corp, Armonk, New York), version 27.0.

## Results

The study population consisted of 13,019 people, comprising 66.1% women and 33.9% men. The average age of the study group was 45.4 years. Within the study group, 14.1% had completed compulsory education, 42.7% had secondary education, and 43.2% had college or university education. The majority (72.7%) were born in Sweden. In total, 25.8% had a mental disorder as their primary diagnosis, 15.5% had a musculoskeletal disorder, and 58.7% had another diagnosis. See Table 1.

**Table 1:** Characteristics of study participants, by having received return-to-work (RTW) coordination or not among patients in primary healthcare

		Received RTW Coordination *n* = 925 (7.1)	Not received RTW Coordination *n* = 12,094 (92.9)	Total *n* = 13,019 (100)
Sex	Female	685 (74.1)**	7,926 (65.6)**	8,611 (66.1)
	Male	240 (25.9)**	4,168 (34.5)**	4,443 (33.9)
Age, years	Median (Md), Mean (SD)	45, 44.5 (10.6)*	46, 45.5 (11.6)*	46, 45.4 (11.5)
Age, in age groups	Up to 29	80 (8.6)**	1,304 (10.8)**	1,384 (10.6)
	30–39	233 (25.2)**	2,694 (22.3)**	2,927 (22.5)
	40–49	292 (31.6)**	3,058 (25.3)**	3,350 (25.7)
	50–59	235 (25.4)**	3,419 (28.3)**	3,654 (28.1)
	60–65	85 (9.2)**	1,619 (13.4)**	1,704 (13.1)
Education level	Compulsory	91 (9.8)**	1,746 (14.4)**	1,837 (14.1)
	Secondary school	396 (42.8)**	5,163 (42.7)**	5,559 (42.7)
	College or university	438 (47.4)**	5,185 (42.9)**	5,623 (43.2)
Country of birth	Sweden	677 (73.2)	8,786 (72.7)	9,463 (72.7)
	Rest of the Nordic countries	26 (2.8)	325 (2.7)	351 (2.7)
	Rest of Europe	65 (7.0)	1,073 (8.9)	1,138 (8.7)
	Rest of world	157 (17.0)	1,909 (15.8)	2,066 (15.9)
Primary diagnosis	Musculoskeletal disorder	133 (14.5)**	1,858 (15.6)**	1,991 (15.5)
	Mental disorder	385 (41.9)**	2,934 (24.6)**	3,319 (25.8)
	Other	400 (43.6)**	7,155 (59.9)**	7,555 (58.7)
Healthcare utilisation, number of healthcare contacts, from March 2019 to February 2020	Md, Mean (SD)	13, 14.7 (9.8)**	6, 8.8 (8.8)**	7, 9.2 (9.0)
Amount of sickness absence (SA) net days, from March 2019 to February 2020	MD, Mean (SD)	121, 135.3 (75.1)**	96, 112.4 (80.2)**	99, 114.0 (80.1)
	< 90 days	270 (29.2)**	5,507 (45.5)**	5,777 (44.4)
	≥ 90 days	655 (70.8)**	6,587 (54.5)**	7,242 (55.6)

The figures are presented as numbers (percentages) if not stated otherwise. Between study participants who had received RTW coordination and those who had not, chi-square tests were used to compare differences in distributions and ANOVA tests were used to compare differences between mean values. *P < 0.05 **P < 0.01

Among the 13,019 people included in this study, only 7.1% had received RTW coordination between March 2019 and February 2020, and the mean number of healthcare contacts was 9.2. The group that had received RTW coordination had a mean of 14.7 healthcare contacts, while the group not receiving RTW coordination had a mean of 8.8 healthcare contacts. See Table 1.

From March 2019 to February 2020, the mean SA days were 114.0. The group that had received RTW coordination had a mean of 135.3 SA days, and the group not receiving RTW coordination had a mean of 112.4 SA days. See Table 1.

### Binary Logistic Regression Models Explaining Received RTW Coordination

Table 2 presents the odds ratios of demographics, diagnosis, healthcare utilisation, SA, and having received RTW coordination from March 2019 to February 2020. In the adjusted logistic regression models, there was an association between being a woman and having received RTW coordination (adjusted OR 1.28, 95% CI 1.09–1.50) compared to men, and also between being in the age group 30 to 39 years (adjusted OR 1.37, 95% CI 1.04–1.80) or the age group 40 to 49 years (adjusted OR 1.55, 95% CI 1.19–2.02) compared to the age group up to 29 years old, and having received RTW coordination. People with higher educational levels were more likely to receive RTW coordination than those with basic education. There was an association between having a secondary school education (adjusted OR 1.37, 95% CI 1.08 to 1.75), or having a college or university education (adjusted OR 1.41, 95% CI 1.10 to 1.79) compared to having compulsory education, and having received RTW coordination. There was no association between country of birth and having received RTW coordination.

**Table 2:** Binary logistic regressions presenting odds ratios (OR) with 95% confidence intervals (CI) for having received return-to-work coordination among patients in primary healthcare

	Crude OR (95% CI)	Adjusted model OR (95% CI)
**Sex**		
Men	Ref	Ref
Women	1.50** (1.29 to 1.75)	1.28** (1.09 to 1.50)
**Age**		
Up to 29	Ref	Ref
30–39	1.41* (1.08 to 1.83)	1.37* (1.05 to 1.80)
40–49	1.56** (1.21 to 2.01)	1.55** (1.19 to 2.02)
50–59	1.12 (0.86 to 1.46)	1.21 (0.92 to 1.59)
60–65	0.86 (0.63 to 1.17)	0.88 (0.64 to 1.23)
**Education level**		
Compulsory	Ref	Ref
Secondary school	1.47** (1.16 to 1.86)	1.37* (1.08 to 1.75)
College or university	1.62** (1.29 to 2.04)	1.41** (1.10 to 1.79)
**Country of birth**		
Sweden	Ref	Ref
Rest of the Nordic countries	1.04 (0.69 to 1.56)	1.26 (0.83 to 1.92)
Rest of Europe	0.79 (0.61 to 1.02)	0.79 (0.60 to 1.04)
Rest of world	1.07 (0.89 to 1.28)	1.06 (0.87 to 1.28)
**Primary diagnosis**		
Other	Ref	Ref
Musculoskeletal disorder	1.28* (1.05 to 1.57)	1.26* (1.02 to 1.55)
Mental disorder	2.35** (2.03 to 2.72)	2.24** (1.92 to 2.61)
**Number of healthcare contacts**	1.05** (1.04 to 1.06)	1.05** (1.04 to 1.06)
**Length of SA period, from March 2019 to February 2020**		
< 90 net days	Ref	Ref
≥ 90 net days	2.03** (1.75 to 2.35)	1.64** (1.41 to 1.91)
Nagelkerke R^2^		0.096

Odds ratio (OR), 95% CI: 95% confidence interval for having received return-to-work coordination during March 2019 and February 2020.

*P < 0.05, **P < 0.01

Adjusted model = Sex + Age + Education level + Country of birth + Primary diagnosis + Number of healthcare contacts + Length of SA period

Receiving RTW coordination was associated with having a musculoskeletal disorder (adjusted OR 1.26, 95% CI 1.02–1.55) or a mental disorder (adjusted OR 2.24, 95% CI 1.92–2.61) compared to other diagnoses. There was an association between a higher number of healthcare contacts and having received RTW coordination (adjusted OR 1.05, 95% CI 1.04–1.06). Having a SA period of 90 net days or more (from March 2019 to February 2020) was also associated with having received RTW coordination (adjusted OR 1.64, 95% CI 1.41–1.91).

### Sickness Absence and Disability Pension in the Ten-Month Follow-Up Period

In the ten-month follow-up period, the mean amount of SA was 71.3 days among people who had received RTW coordination from March 2019 to February 2020. The corresponding number for people who had not received RTW coordination was 76.1 SA days. See Table 3. The ANOVA showed no statistically significant differences in the taken SA days in the follow-up period (*p*-value = 0.118) between those who had received RTW coordination and those who had not. When DP was also included in the follow-up period, the mean amount of SA/DP was 71.4 days among people who had received RTW coordination during the previous period and 76.4 days for those who had not. In the follow-up period, ANOVA showed no statistically significant differences in the amount of SA/DP days (*p*-value = 0.105), or in exclusively DP days (*p*-value = 0.453) between the people who had received RTW coordination and those who had not.

**Table 3:** Mean days of sickness absence (SA), disability pension (DP) and SA/DP in the ten-month follow-up period, distributed on having received return-to-work coordination or not from March 2019 to February 2020 among patients in primary healthcare

	Received RTW coordination		Not received RTW coordination		Total		ANOVA between groups
	Md	Mean (SD)	Md	Mean (SD)	Md	Mean (SD)	
SA	34	71.3 (88.1)	39	76.1 (91.3)	39	75.8 (91.1)	0.118
DP	0	0.1 (4.0)	0	0.3 (7.5)	0	0.3 (7.3)	0.453
SA/DP	34	71.4 (88.4)	40	76.4 (91.5)	39	76.1 (91.3)	0.105

*Md* Median, *SD* Standard deviation

The amount of SA and the amount of SA/DP in the ten-month follow-up period were used as outcomes in negative binomial regression analyses. In these analyses, received RTW coordination was not associated with SA days in the ten-month follow-up period, neither in the unadjusted (IRR = 0.94, 95% CI: 0.88–1.00) nor in the adjusted model (adjusted IRR = 0.96, 95% CI: 0.90–1.03). However, received RTW coordination was associated with fewer SA/DP days in the unadjusted negative binomial regression model (IRR = 0.93, 95% CI: 0.87–1.00), but the association vanished in the adjusted model (adjusted IRR = 0.96, 95% CI: 0.90–1.03). See Table 4.

**Table 4:** Negative binomial regression model explaining the amount of sickness absence (SA) and SA/disability pension (DP) in the ten-month follow-up period among patients in primary healthcare

	Unadjusted IRR			Adjusted IRR		
	Amount of SA days in the ten-month follow-up period			Amount of SA days in the ten-month follow-up period		
	IRR	95% CI	*P*-value	IRR	95% CI	*P*-value
Received RTW coordination from March 2019 to February 2020						
No	Ref			Ref		
Yes	0.94	0.88 to 1.00	0.053	0.96	0.90 to 1.03	0.300

	Amount of SA/DP days in the ten-month follow-up period			Amount of SA/DP days in the ten-month follow-up period		
	IRR	95% CI	*P*-value	IRR	95% CI	*P*-value
Received RTW coordination from March 2019 to February 2020						
No	Ref			Ref		
Yes	0.93	0.87 to 1.00	0.045	0.96	0.90 to 1.03	0.280

Incidence Rate Ratios (IRR), confidence interval (CI) and P-value for the amount of SA and SA/DP days in the ten-month follow-up period (from March 2020 to December 2020)

Adjusted binomial regression model = Return-to-work (RTW) coordination from March 2019 to February 2020 + Sex + Age + Education level + Country of birth + Primary diagnosis + Number of healthcare contacts + SA days from March 2019 to February 2020

## Discussion

The results of this study show that receiving RTW coordination in a primary healthcare clinic is associated with being a woman, being 30 to 49 years of age, and having a higher level of education, more healthcare contacts, more SA days, and a musculoskeletal or mental disorder. These results might be driven by the fact that most primary healthcare RTWCs prioritise patients they perceive as at risk of a lengthy SA [14, 15]. Another study indicated that most of the patients who come into contact with an RTWC are referred by some other healthcare professional and only a relatively small proportion find their way to a RTWC autonomously [5]. The results in this study imply that healthcare professionals work in line with RTWC priorities and refer patients to RTWCs if they notice a risk for long-term SA and a pronounced need for support in the RTW process.

Mental and musculoskeletal disorders are frequent causes of long-term SA, especially among women [20, 21]. In line with previous studies [5, 14], this study indicates that people with common mental disorders receive RTW coordination more often than those with other diagnoses. The higher number of healthcare contacts and SA days among people with mental and musculoskeletal disorders also indicates that people who received RTW coordination were more likely at a higher risk for long-term SA than those with other diagnoses. Different healthcare seeking patterns, where men more often than women tend to avoid or delay seeking healthcare [22] might also account for the distinctive characteristics of the group receiving RTW coordination. Even though RTWCs are instructed to promote gender equality in their work with the RTW process [23], they are to a large extent dependent on the gender sensitivity of the healthcare professionals who refer patients to them.

Research findings are not unanimous about the effect of RTW coordination on RTW outcomes. Some studies demonstrate that RTW coordination interventions have a positive effect on RTW [7–9], while others have found no, or even adverse, effects [10, 11]. Some research also suggests that interventions involving a RTWC may be more time-consuming than other approaches [4, 6]. In the adjusted analyses, this study did not show an association between RTW coordination in primary healthcare and the amount of SA taken in the following ten-month period. Previous research has suggested that there might be a time-lag effect with RTW coordinating interventions [24] and that the benefits in terms of RTW only materialise slowly, which may explain the weak association seen in this study. Another explanation for the weak association between RTW coordination and RTW outcome might be the distortive effect of confounding by indication [25]. Confounding by indication is likely to occur when a particular intervention, such as RTW coordination, is linked to the outcome, such as RTW. The results show that people who received RTW coordination may differ from those who did not due to factors associated with long-term SA, such as being a woman [26], the type of diagnosis (such as psychiatric or pain disorders [20, 21, 27]), and the number of healthcare visits [28]. Hence, it is reasonable to assume that people who receive RTW coordination are considered more likely to have longer durations of SA. People with less challenging RTW trajectories may also have been opted out from an RTW coordination intervention, an action which may contribute to this study’s weak association between RTW coordination and SA days. Although lower educational level is a known risk factor for long-term SA [29], in this study receiving RTW coordination was associated with higher educational attainment. The higher levels of unemployment among people with lower educational attainment [30] might signal that unemployed people on SA do not receive RTW coordination to the same extent as those in paid work. The associations between unemployment, SA, and access to RTW coordination have not been studied here because our data lacked this information; more research in this area is warranted.

The results of this study do not show the group receiving RTW coordination taking more SA days in the following ten-month period than those who did not, even though it is reasonable to assume that this group, to some extent, are prone to have a higher risk for long-term SA. Instead, the results show a non-significant reduction of approximately five days less SA in the following ten-month period for the group who received RTW coordination. However, even a small reduction in SA days may be substantial from a population perspective since any reduction in SA means a social cost saving [31, 32]. A recently published Finnish study showed that RTW coordination would be cost saving if it decreased SA by at least 2 days in comparison to regular care with no intervention [31]. Assuming that the cost for RTW coordination in Sweden is likely similar, a reduction of approximately five days in SA and SA/DP for RTW coordination receivers could justify the resource allocation to RTW coordination. Further, randomised controlled studies are needed to better determine and estimate RTW coordination effects on RWT outcomes.

Previous research has found that perceived adequacy of professional support may influence RTW [33]. RTWCs were implemented to improve the quality of the interaction between people on SA, the healthcare services and other stakeholders [34]. This register-based study cannot judge the quality of the interactions between people on SA and the professional who provided their RTW coordination. However, by only including clinics with an employed RTWC, of whom nearly all had had professional training and had completed university courses in the coordination of rehabilitation and the RTW process [35], it is reasonable to assume that the coordination they provided about RTW was of a sufficiently high quality. Studies of people who met a primary healthcare RTWC during the same time period and in the same region also report positive experiences of the RTWC interventions [5, 34, 36].

## Strengths and Limitations

The strengths of this study include the use of register data, which is considered to be of high quality. There are also limitations. Measuring the characteristics of the people on SA who had received RTW coordination was based on a single time period. Therefore, it is not possible to determine the temporal or cause-effect relationship between the RTW coordination intervention and associated measures. It is possible that some unmeasured, but important confounders, were not included in the multivariate analysis to assess the adjusted association between the explanatory measures and the outcomes. Another limitation was that the data were not processed so that the first follow-up dates were analysed in relation to the exact date when each individual received their RTW coordination. However, from the comparative point of view of the study, it is not possible to use exact dates, as the corresponding “start date” does not exist for those individuals who did not receive RTW coordination. Although the study has the limitation of not analysing exact follow-up dates at the individual level, there is a strength in that it is based on a large amount of data, and we can show group-level estimates based on time periods. Employment status may influence whether an individual receives SA and RTW coordination, and a further limitation, was that no information on employment status was available for analysis in the study.

RTW coordination can include a variety of tasks and interventions, such as planning the RTW, supporting the person on SA, and collaborating with that person’s manager during the SA period [6, 12, 15, 37]. In this study, indicated RTW coordination was based on the registered intervention code for RTW coordination, GC001, which does not disclose any information on the specific activities a RTW coordination included. In addition, because both RTWCs and other healthcare professionals may deliver RTW coordination and use the intervention code for RTW coordination, it was not possible to distinguish if the code was registered by a RTWC or not, although the decision to include only clinics with an employed RTWC during the period from March 2019 to February 2020 was taken to minimise the chance of an RTW coordination being delivered by someone other than a RTWC. It would be unusual for other healthcare professionals to have knowledge about the intervention code for RTW coordination, although they may help to coordinate with, for example, the Swedish Social Insurance Agency or the patient’s employer. This code is thus used only sporadically by others apart from RTWCs, which is why the content and the quality of RTW coordination at clinics without an RTWC can vary significantly. Some RTWCs did not systematically register intervention codes during the study period.

The generalisability of the findings may be limited to countries with a similarly organised RTW coordination process within primary healthcare. There is a need to further investigate the factors associated with receiving RTW coordination and its potential outcomes. There is also a need for future research to evaluate the specific interventions and components included in the RTW coordination and to obtain a more in-depth understanding of which components have the potential to decrease SA and which do not. Analyses of the effect of RTW coordination in different subgroups, such as gender or employment status, are also desirable in future studies.

## Conclusion

This study shows that people on SA who receive RTW coordination in primary healthcare clinics might differ from those who do not receive RTW coordination, especially regarding factors that have been shown to predict a risk of long-term SA. This indicates that RTW coordination seems to be directed towards groups of people with a higher risk of long-term SA. However, other people with long-term SA did not receive RTW coordination, which raises the question of whether or not more people on SA would have benefited from RTW coordination as well. This would likely mean that more finely calibrated ways of identifying people at risk of long-term SA are needed. In addition, the results showed a non-significant net benefit during the following ten months of approximately five fewer days of SA for the group who received RTW coordination in primary healthcare. Future studies are needed to establish if RTW coordination significantly decreases SA days.

## Data Availability

The data cannot be made publicly available due to privacy regulations. According to the General Data Protection Regulation, the Swedish Data Protection Act, the Swedish Ethical Review Act, and the Swedish Public Access to Information and Secrecy Act, data can only be made available for specific purposes, including research that meets the criteria for access to this type of sensitive and confidential data as determined by a legal review. We used data from the REWHARD consortium supported by the Swedish Research Council (grant no. 2021–00154).
